# Long Non-Coding RNA (lncRNA) Roles in Cell Biology, Neurodevelopment and Neurological Disorders

**DOI:** 10.3390/ncrna7020036

**Published:** 2021-06-17

**Authors:** Vincenza Aliperti, Justyna Skonieczna, Andrea Cerase

**Affiliations:** 1 Department of Biology, University of Naples Federico II, 80126 Naples, Italy; 2 Centre for Genomics and Child Health, Blizard Institute, Barts and The London School of Medicine and Dentistry, Queen Mary University of London, London E1 2AT, UK; j.b.skonieczna@smd19.qmul.ac.uk

**Keywords:** long non-coding RNAs, neurodevelopment, neurological disorders, neurodegeneration, neuropsychiatric disorders, Alzheimer’s disease (AZ), amyotrophic lateral sclerosis (ALS), autism spectrum disorder (ASD), schizophrenia (SZ)

## Abstract

Development is a complex process regulated both by genetic and epigenetic and environmental clues. Recently, long non-coding RNAs (lncRNAs) have emerged as key regulators of gene expression in several tissues including the brain. Altered expression of lncRNAs has been linked to several neurodegenerative, neurodevelopmental and mental disorders. The identification and characterization of lncRNAs that are deregulated or mutated in neurodevelopmental and mental health diseases are fundamental to understanding the complex transcriptional processes in brain function. Crucially, lncRNAs can be exploited as a novel target for treating neurological disorders. In our review, we first summarize the recent advances in our understanding of lncRNA functions in the context of cell biology and then discussing their association with selected neuronal development and neurological disorders.

## 1. Introduction

For a long time, scientists believed that functional genetic information was only contained in protein-coding genes. Proteins were considered the main protagonists in cellular functions, while RNAs were thought to be mere intermediaries between DNA and proteins [1].

In recent years, advances in genomic sequencing technology and findings from large-scale consortia have facilitated our understanding of the mammalian genome’s complexity and flexibility. Indeed, genome-wide analyses of the eukaryotic transcriptome have shown that about 90% of the human genome is actually transcribed. Only about 2% of it is annotated as protein-coding genes (ENCODE Project Consortium, https://encodeproject.org, v117, accessed on 28 May 2021) [2], while the majority of transcripts represent non-coding RNAs (ncRNAs) [3]. ncRNAs are a heterogeneous group of genes that do not have functional open reading frames (ORFs) and are not transcribed into proteins. Due to these biological characteristics, for a long time, they were considered “junk” [4,5]. Recent data have shown that this part of the genome is functionally critical and is involved in physiological processes and tissue homeostasis [6,7] both in health and disease [8,9,10,11,12,13,14,15,16,17,18]. This has also been supported by the evolutionary analysis of conserved ncRNAs [8]. Indeed, while the number of protein-coding genes has remained relatively stable, the number of non-coding transcripts have increased considerably in parallel with the complexity of organisms [19]. In this review, we discuss the diverse, general mechanisms of action of well-studied lncRNAs and then focus on the role of lncRNAs in selected, primary examples of neurodevelopmental and neurological disorders.

### 1.1. Classification of ncRNAs

**Figure 1:**
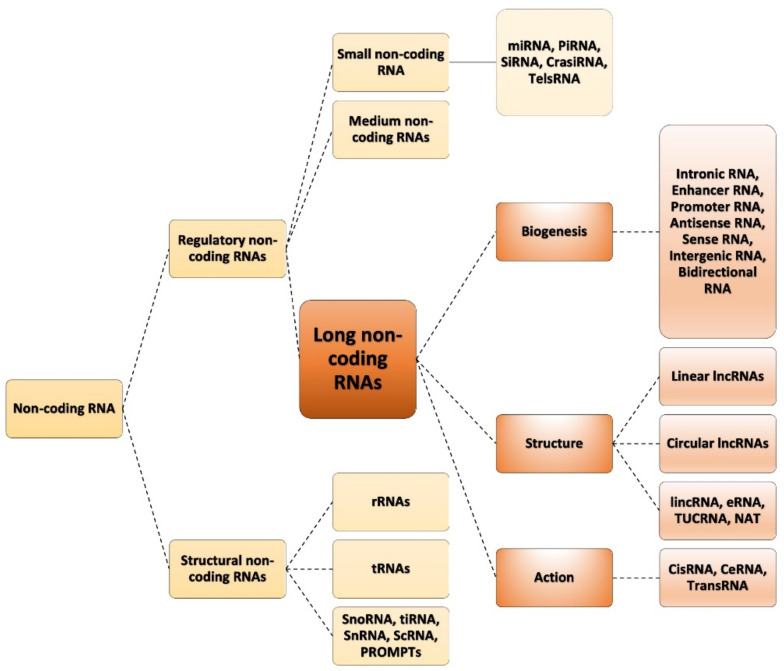
ncRNAs classes: schematic classification of ncRNAs into classes and sub-classes according to their actions, biogenesis and structures (snoRNA: small nucleolar RNAs; tiRNA: transcription initiation RNA; snRNA: small nuclear RNA; scRNA: small cytoplasmic RN; PROMPTs: promoter upstream transcripts; rRNAs: ribosomal RNAs; tRNAs: transfer RNAs; miRNA: micro RNA; piRNA: piwi RNA; siRNA: small interfering RNA; crasiRNA: centromere repeat-associated small interacting RNA; telsRNA: telomere-specific small RNA; lincRNA: long intergenic noncoding RNA; eRNA: enhancer-derived RNA; TUCRNA: transcribed ultraconserved RNA; NAT: natural antisense transcript; *cis*-lncRNA: *cis*-acting long non-coding RNA; ceRNA: competing endogenous RNA; *trans*-lncRNA: *trans*-acting long non-coding RNA).

NcRNAs may be grouped into different classes and classified according to size and function. In particular, ncRNAs are divided into two main classes: structural ncRNAs and regulatory ncRNAs (Figure 1) [20]. The former are usually considered constitutive and include ribosomal RNAs (rRNAs), transfer RNAs (tRNAs), small nuclear RNAs (snRNAs) and small nucleolar RNAs (snoRNAs). Regulatory ncRNAs, on the other hand, are in turn divided based on their length into three classes: short ncRNAs, which include microRNAs (miRNAs, 22–23 nucleotides (nts)) and piwiRNAs (piRNAs, 26–31 nts); medium ncRNAs (50–200 nts); and long ncRNAs (lncRNAs, > 200 nts) [21,22]. Most of these transcripts are generated by post-transcriptional cleavage. These can be small or large RNA fragments, which function independently from one another upon cleavage [23].

### 1.2. Biogenesis of lncRNAs

**Figure 2:**
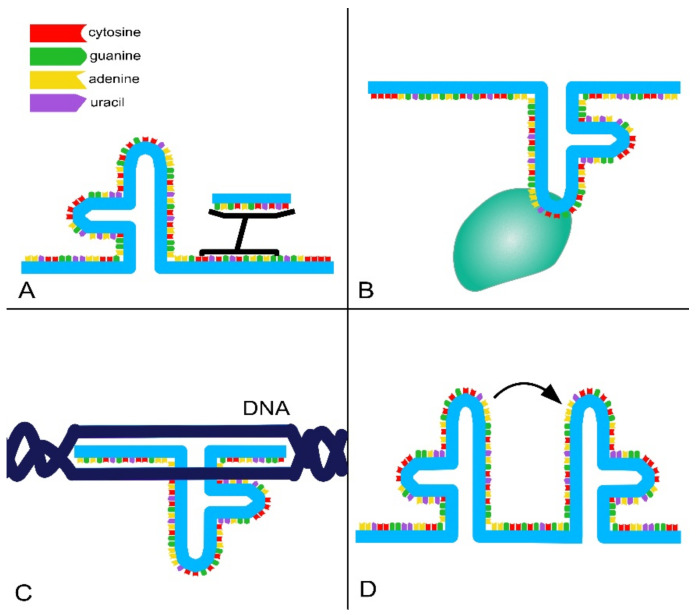
Domain architecture of lncRNAs. Schematic representation of lncRNAs’ structural domains, through which they can bind other RNAs via complementary base–pair interactions (**A**), proteins (**B**) and DNA (**C**). These interactions can induce allosteric, conformational changes to other structures in the lncRNA (**D**).

A significant part of non-coding transcripts is represented by lncRNAs, which are RNA transcripts longer than 200 nts. The biogenesis of lncRNAs resembles that of messenger RNAs (mRNAs). They are transcribed by polymerase II (Pol II) and can be polyadenylated, spliced and 5′-capped [7,21,23]. The mechanisms involved in the biogenesis of lncRNAs are cell type-specific and controlled by stage-specific stimuli [20]. They undergo post-transcriptional modifications and inter/intra-cellular transport. Most of them have different expression patterns and preferentially nuclear localization, in contrast to mRNAs, because they are involved in chromatin and epigenetic regulation of gene expression [2,7,21,24]. Once transcribed, lncRNAs fold into a thermodynamically stable secondary structure. RNA has the ability to form double helices, hairpins and pseudoknots thanks to high-level tertiary interactions, mainly mediated by couplings of non-canonical bases. As shown in Figure 2, four functional domains can be present in lncRNAs [25]:

- RNA-binding domains. Thanks to their ability to base pair with other RNAs, lncRNAs can recognize and bind mRNAs, miRNAs and other lncRNAs, modulating target levels and function;
- Protein-binding domains. Proteins are a major partner of lncRNAs, forming ribonucleoprotein complexes (RNPs) that act as chaperones, transport aids or effectors (including phase-separation seeding). This type of interaction involves conformational changes in the protein, RNA or both;
- DNA-binding domains. Currently, there is a lack of extensive evidence for direct and functional interaction between lncRNAs and DNA as well as a lack of a consensus regarding the role and function of these interactions. However, it is known that RNA–DNA hybrids or triplex structures can allow single strands of RNA to interact with DNA duplexes through pair–base interactions. These direct interactions can efficiently and selectively direct RNA signals to genomic loci through base-pairing interactions. However, such interactions can also expose the genome to deamination and damage;
- Conformational switch. LncRNAs can act as regulatory devices by allosterically coupling binding domains with the switching of structural conformations and thereby activating or suppressing linked functional domains.

### 1.3. Types of lncRNAs

**Figure 3:**
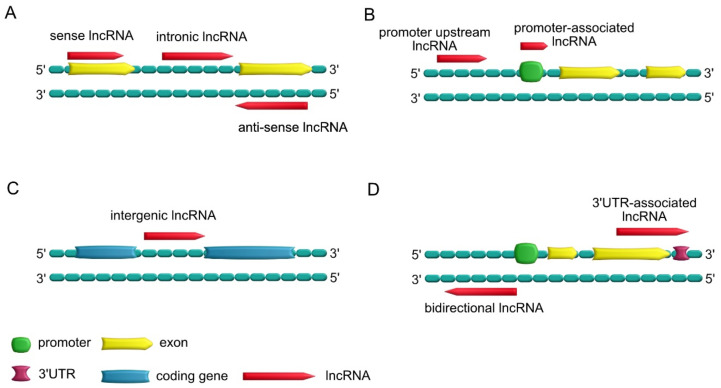
Classification of lncRNAs. (**A**) Sense lncRNAs and antisense lncRNAs are, respectively, present on the same and opposite strands and overlap with neighboring mRNAs; intronic lncRNAs are transcribed from the introns of the protein-coding genes. (**B**) Promoter upstream lncRNAs are located upstream of the promoter; promoter-associated lncRNAs are close to the promoter. (**C**) Intergenic lncRNAs are transcribed from the genomic interval between two genes. (**D**) Bidirectional lncRNAs have transcription start sites that are close to adjacent genes on the antisense strand, and these have common promoters; 3′UTR-associated lncRNAs are obtained from a protein-coding gene’s 3′UTR region.

LncRNAs can be classified based on their biogenesis, structure, localization or mechanism of action [20,26,27]. Based on their localization in the genome compared to protein-coding genes, they can be further divided into various subclasses (Figure 3) [28]:

- Sense lncRNAs, which overlap one or more exons of neighboring mRNAs on the same strand; antisense lncRNAs, which overlap one or more exons of neighboring mRNAs on the opposite strand; intronic lncRNAs, which are transcribed from introns of a second transcript (sometimes may represent pre-mRNA sequences);
- Promoter upstream lncRNAs, which are located upstream of a promoter; promoter-associated lncRNAs, which are close to the promoter;
- Intergenic lncRNAs, which lie within the genomic interval between two genes;
- Bidirectional lncRNAs, which have promoters in common with protein-encoding genes but are transcribed in the opposite direction; 3′UTR-associated lncRNAs, which are transcribed from a protein-coding gene’s 3′UTR region.

Another approach in the classification of lncRNAs is based on their subcellular localization, which can be linked to their function (such as chromatin-associated lncRNAs, chromatin-interlinking RNAs, nuclear bodies-associated RNAs and PRC2-associated RNAs [29]) or to their structure, distinguishing linear lncRNAs from the circular lncRNAs (circRNAs that are produced in a process called “back-splicing” [30].

Finally, based on the mode of action, lncRNAs can be divided into *cis*-acting lncRNAs, *trans*-acting lncRNAs (i.e., working on the same chromosome they are transcribed for, or not, respectively) and competing endogenous lncRNAs, which share sequence and function similarities with mRNAs and compete with them for function [20,29].

### 1.4. Evolution, Conservation and Stability of lncRNAs

Comparing lncRNAs with protein-coding genes and small ncRNAs, lncRNAs were found to be poorly conserved at the primary structure level (i.e., sequence conservation). In fact, for protein-coding genes, the average number of nucleotide substitutions is approximately 10%, while for lncRNAs, this number increases up to 90–95%, thus estimating that only 5–10% of the sequences are preserved [31]. A noticeable example is *Xist*, the lncRNA involved in the inactivation of one of the two X chromosomes in mammalian females, which appears to be relatively poorly conserved in some eutherians’ clades [32]. Multidisciplinary studies have, however, highlighted how regions such as the promoter and the exon–intron boundaries are much more conserved in lncRNAs sequence [33]. Furthermore, in a recent work, Kirk and colleagues [34] developed the SEEKR (sequence evaluation through k-mer representation) method, which allows quantifying the similarity of nonlinear sequences between lncRNAs by evaluating all possible sequence combinations at a given length (k) within the lncRNA. In this way, it is possible to expand the number of significant correlations with protein binding and lncRNA subcellular localization. For example, substantial levels of nonlinear sequence similarity were found between functional domains in *Xist* and domains in the *Rsx* lncRNA, a marsupial lncRNA originating from convergent evolution that has been proposed as a functional analogue of *Xist* [35,36]. Therefore, k-mer represents a promising approach for the functional classification of lncRNAs based on their sequence [36].

Sequence analysis and experimental investigation also allowed obtaining information on the lncRNAs’ secondary structure. LncRNA secondary structures are characterized by modular structures, organized in independent RNA modules with different functions. Each domain contains several structural motifs such as internal and terminal loops as well as helices and junction regions regulating its function. [37]. To date, few lncRNAs have been characterized at the secondary structure level including *Xist* [37,38,39,40,41], *HOTAIR* [37,42], lincRNA-*p21* [37,43] and several others. For instance, *HOTAIR* consists of four independent domains containing 56 helical segments, 38 terminal loops, 34 internal loops and 19 junction regions [37,42]; *Xist* is characterized by different conserved regions of tandem repeats, which are indispensable for specific functions [44]. For example, its 5′ region is highly conserved and consists of 8.5 copies in humans (7.5 in mice) of 26-mers separated by U-rich linkers (A-repeat) that assume double stem–loop structure ensembles, which function as a platform for the protein binding involved in gene silencing [44]. All these structures can undergo rearrangements due to polymorphisms or post-transcriptional modifications that alter their stability. It is therefore deduced that lncRNAs do not strictly have a conserved primary structure but maintain conserved functions that are carried out through the promoter regions, splicing patterns, expression patterns and secondary structure through interaction with RNA-binding proteins [26,45].

Stability is an important feature for the functional analysis of lncRNAs. It is, in fact, known that the average half-life of each RNA is related to its physiological function. LncRNAs have a much shorter half-life and greater variability than mRNAs. In particular, it is possible to divide lncRNAs into unstable (average half-life < 2 h), stable (average half-life > 2 h) and extremely stable (average half-life > 16 h). Furthermore, intergenic and antisense lncRNAs are more stable than intronic ones, just as the transcripts that have undergone splicing are more stable than those that have not undergone it (single exon). Finally, nuclear lncRNAs are probably more unstable than others with different subcellular locations [46].

## 2. Functional Roles of lncRNA in Cellular Processes

LncRNAs are dynamically expressed during cell differentiation and development. They are able to regulate the cell cycle, genetic imprinting and stem cell reprogramming [26,45,46,47,48,49,50,51]. An increasing number of lncRNAs are specifically expressed during brain development as well as neural stem cell and progenitor differentiation. Some examples are *MALAT1* (metastasis-associated lung adenocarcinoma transcript 1), which regulates synaptogenesis, and *Sox2OT* (*Sox2* overlapping transcript), which overlaps the *Sox2* gene encoding for a transcription factor important for neural stem cell self-renewal [52]. LncRNAs play a key role in the development and onset of several related neuropathologies such as Down syndrome, Rett syndrome, Fragile X syndrome and autism spectrum disorders, wherein abnormal expression of lncRNAs affects neurodevelopment and plays a major role in pathogenesis [3,52,53,54,55,56,57].

The main biological functions of lncRNAs include epigenetic regulation, chromatin remodeling and protein metabolism control. They can act at the transcriptional and post-transcriptional level, *in cis* or *trans*, and also act as a signaling molecule with a scaffolding role [20,28,50]. By definition, lncRNAs are different from coding mRNAs because they lack a substantial ORF and fail to produce proteins [26,58,59,60]. However, recent evidence has shown that some annotated lncRNAs can actually encode small functional peptides [61]. In addition, a recent analysis by Ruiz-Orera and colleagues [62] on ribosome profiling experiments provided important evidence that lncRNAs associated with ribosomes might play an important role in de novo protein evolution by encoding short peptides. The role of these peptides is not yet known and if they are functional. It is therefore believed that exploring the pathological and physiological effects of the new peptides generated by lncRNAs can unlock new fields of investigation.

### 2.1. Mechanisms of Action

LncRNAs are very versatile molecules that have the ability to create physical and functional interactions with DNA, RNA and proteins through base pairing or through functional domains, which are generated thanks to their secondary and tertiary folding (discussed above) [63].

LncRNAs can regulate gene expression positively or negatively through multiple mechanisms. Many lncRNAs act via chromatin modulation by working as molecular scaffolds for protein–protein interactions or interacting with chromatin-modifying complexes and recruiting chromatin-modifying complexes at specific loci to activate or repress target gene expression [64,65]. Some lncRNAs affect transcription by modulating the binding of the general transcription machinery and regulatory factors [4,29,65,66,67]. They are involved in RNA processing [65,68], RNA turnover, silencing, translation and decay of mRNAs [49,65,69,70] or act as miRNA sponges to neutralize miRNA-mediated mRNA silencing [65,71]. In addition, some lncRNAs are determined to be precursors of certain miRNAs at particular stages of development [30,65].

The function of lncRNAs also depends on their subcellular localization. They can localize in different cellular compartments including the nucleus, the chromatin and the cytoplasm based on sequence and structural motifs [72]. For example, the AGCCC motif is strongly correlated with lncRNAs nuclear localization [73]. Therefore, more and more resources have been spent on the design of tools that allow predicting lncRNAs sub-cellular location using nucleotide compositions [72,74]. Many are important modulators for nuclear functions. Several lncRNAs act *in cis* on their transcription sites. In this way, they manage to modulate local gene expression, both by recruiting transcription factors and/or chromatin modifiers and might be forming a DNA–RNA triplex that anchors lncRNA and the effector proteins associated with the gene promoter [20]. Others need to be relocated from their synthesis sites while maintaining nuclear localization. They, therefore, act *in trans* to have an impact on gene regulation. Finally, other lncRNAs must be exported to the cytoplasm to play their regulatory roles, interfering with post-translational modifications or influencing gene regulation by acting as decoys for miRNAs and proteins [20,28,50].

### 2.2. LncRNAs as Chromatin Regulators

LncRNAs are involved in global epigenetic reprogramming during cell growth and development. Within the nucleus, they can affect chromatin status through inter- and intra-chromosome interactions, remodeling chromatin and its condensation by acting on specific chromatin loci and regulating gene expression through mechanisms such as methylation or acetylation without changing the DNA sequence. Additionally, lncRNAs influence chromosome bridging by binding to heterogeneous nuclear ribonucleoprotein U [3,5,28,64]. A fraction of lncRNAs bind to the Polycomb repressive complex 1/2 (PRC1/2) [75] or the chromatin-modifying proteins CoREST and SMCX [76]; others bind to trithorax chromatin-activating complexes (trxG) [77]. These complexes act as antagonists in gene expression regulation during cell development and differentiation. In particular, while the PRC2 complex plays a role in repressive histone modifications, trxG promotes the transcriptional activation of *Hox* genes [78,79].

The well-characterized lncRNAs *HOTAIR*, *ANRIL*, *XIST* and *KCNQ1OT1* are able to recruit epigenetic modifiers to specific loci for reprogramming the chromatin state [64,80]. For example, *HOTAIR* acts as a scaffold for coordinating the targeting of specific repressive, histone-modifying complexes to target loci [80]; *XIST* mediates X chromosome inactivation by recruiting repressive histone complexes such as PRC1 and PRC2 [81,82,83] and DNA-methylating complex [84]. Additionally, lncRNAs affect allelic gene expression through imprinting. Gene expression is regulated by specific genomic loci where protein-coding genes and lncRNAs are reciprocally expressed (Nesp/Nespas, Igf2r/Air, Dlk1/Gtl2). Moreover, some lncRNAs in specific loci may also control the imprinting regulation of neighboring genes via additional epigenetic factors [7].

### 2.3. Transcriptional, Post-Transcriptional and Post-Translational Regulation

The regulation of transcription by lncRNAs is mediated through chromatin regulation and various transcription factors (TFs), modifying lncRNAs activity and/or binding [28,49,64]. In particular, some lncRNAs regulate transcription via competing for TFs or recruiting TFs acting as either co-activators or co-repressors of specific genes [7,28,49,85,86,87,88]; others lncRNAs directly act on RNA polymerase II by interacting with the initiation complex [89,90].

LncRNAs play a key role also in post-transcriptional events such as mRNA splicing, editing, transport, translation and degradation [28,30,64]. For instance, several nuclear lncRNAs including *NEAT1*, *MALAT1*, *MIAT* (also known as *GOMAFU*) and *SAF* were linked to splicing regulation. They recognized splicing factors and influenced their activity by either modulating their post-translational modifications (e.g., phosphorylation) or by regulating interactions with other splicing factors and with protein-coding mRNAs [19,91,92,93,94]. Furthermore, lncRNAs can be implicated in alternative splicing through lncRNA-mediated chromatin remodeling [92,93]. For example, *MIAT* binds splicing factor 1 (SF1) protein through its UACUAAC repeat sequences and inhibits splicing and spliceosomal complex formation [95]; *MALAT1* regulates the alternative splicing of endogenous target genes through the modulation of the phosphorylation status of splicing factors [68,96].

LncRNAs can also regulate RNA levels. Some of them can alter their stability by acting on the 3′UTR regions rich in AU elements, with consequences for transcript degradation, decapping and deacetylation [7,49,69]; others such as *BACE1AS* can act to form an RNA–RNA duplex, increasing the stability of the mRNA [7,49,97].

LncRNAs can also act as scaffolds for higher-order complexes. For examples, lncRNAs can organize translational particles during ribosome translation in the endoplasmic reticulum (ER) [36] and to mediate stress granule formation through RNA–RNA interactions [5]. Finally, they can interact synergistically with mRNAs and act as miRNA inhibitory regulators modulating target expression [28,30,64,98,99,100].

Finally, lncRNAs play a role in the post-translational modifications of proteins including phosphorylation, ubiquitination and acetylation. Therefore, they regulate protein formation, degradation and expression [101]. For example, in cancer, the lncRNAs *HULC* promotes cell proliferation through activation of the ERK pathway, with consequent phosphorylation of YB-1 preventing its interaction with other oncogenic mRNAs [102].

## 3. LncRNAs in Neurological and Neurodegenerative Disorders

LncRNAs are involved in the cell differentiation and function of all cell lineages. Given the many functions of lncRNAs, it is important to understand that any mutation resulting in either gain- or loss-of-function can have a major impact on protein synthesis, metabolism and many other cellular activities. In this review, we will focus on selected examples of neurological and neurodevelopmental disorders (see below).

Several pieces of evidence have shown that lncRNAs dysregulation is related to various neurological disorders including neurodegenerative diseases and neuropsychiatric disorders [50,65]. Neurodegenerative disorders are characterized by progressive neuron dysfunction and/or degeneration, affecting the nervous system functionality. The consequent clinical symptoms, both motor and cognitive, vary and are characteristic of the specific disorder. Moreover, neurodegenerative disorders can be age-related or non-age-related and sex-biased and be either familiar or sporadic [4,26,31,103,104]. LncRNAs play a significant role in the pathophysiology of these disorders due to their important regulatory functions within the cell, involvement in various signalling pathways and the functioning of mitochondria; hence, their dysregulation may lead to the death of neurons and brain atrophy (Table 1) [26,105,106]. Therefore, it is crucial to expand our knowledge on the lncRNAs involved in the pathophysiology of these diseases and their mechanisms of action. In this way, we will be able to gain a better understanding of the processes underlying neurological diseases (for example, the signaling pathways involved) in order to create better diagnostic tools and new forms of treatments [106].

For this review, we selected noticeable examples of lncRNA role in diseases such as Alzheimer’s disease (AD) and amyotrophic lateral sclerosis (ALS). Finally, as prime examples of defective neurodevelopment, we chose to discuss the role of lncRNA in schizophrenia (SZ) and autism (ASD).

### 3.1. Alzheimer’s Disease

AD is one of the most prevalent aging-related neurodegenerative diseases and causes of dementia worldwide [109,131,132]. This disease is characterized by progressive degeneration of cortical neurons, leading to brain tissue atrophy and clinical symptoms such as dementia and cognitive decline. The two main characteristics of AD are the accumulation of amyloid-beta (Aβ) in the extracellular senile plaques and hyperphosphorylated tau protein in the intracellular neurofibrillary tangles [26,106,131,132,133,134]. However, several other factors can cause neurodegeneration such as neuroinflammation and oxidative stress [131].

AD is a multifactorial disease associated with several risk factors. It has a strong genetic component, with mutations in genes responsible for Aβ processing linked to the pathophysiology of this disease. Research from recent years has shown that lncRNAs play a major role in AD through epigenetic control of target genes (Table 1) [135,136,137,138]. Studies evaluating the profiles of aberrantly expressed transcripts in AD animal models showed that most of the lncRNAs upregulated or downregulated in AD were linked to metabolic pathways (in some cases, through insulin signalling), inflammatory processes and also synaptic transmission [136,139]. Interestingly, some lncRNAs that are dysregulated in AD have an opposite expression profile in cancer. For instance, the transcripts linked to neurodegeneration that are downregulated in AD are instead upregulated in cancer and involved in processes associated with the survival and proliferation of cancer cells [136,140].

**Figure 4:**
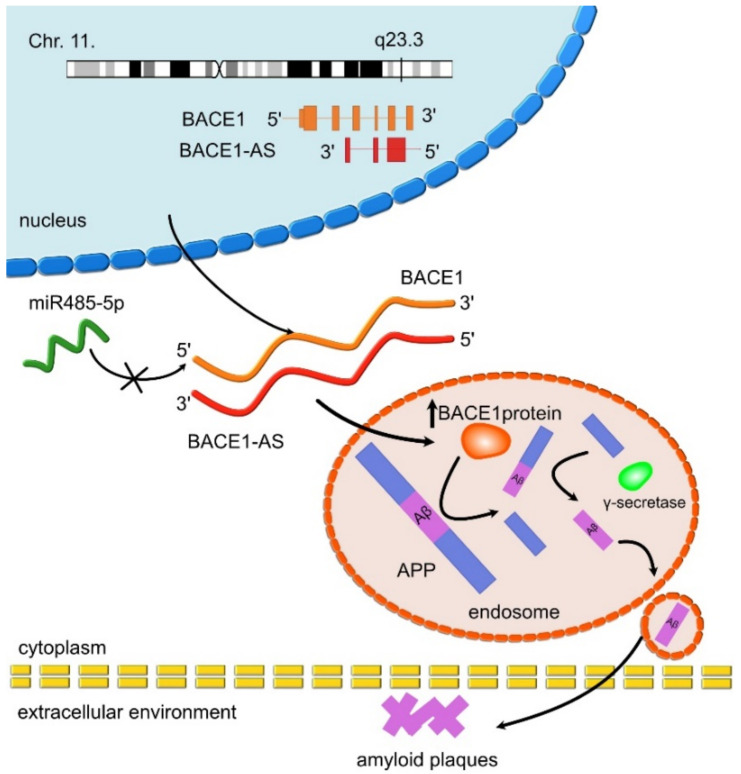
*BACE1-AS* involvement in AD pathophysiology. The *BACE1-AS* locus is localized on chromosome 11 (www.genecards.org, accessed on 28 April 2021). Its product binds to *BACE1*, stabilizing this mRNA and masking a binding place for miR485-5p, which normally inhibits the *BACE1* enzyme translation. In this way, BACE1 leads APP into the amyloid pathway, increasing the production of the Aβ aggregates into the amyloid plaques.

One of the best-studied lncRNAs transcripts to date is *BACE1-AS* (Table 1), which is the antisense transcript to the gene encoding beta-secretase 1 (*BACE1*), which is involved in the amyloid pathway of Aβ cleavage. In particular, the enzyme encoded by *BACE1* is responsible for the cleavage of the amyloid precursor protein (APP). *BACE1* overexpression results in the increased synthesis of the misfolded protein. Therefore, BACE1 levels can be used as a blood plasma biomarker for brain amyloidosis in people with AD [137,141]. *BACE1-AS* expression upregulates the transcription of *BACE1* mRNA through the formation of stabilizing RNA duplex by binding to the open reading frame of BACE1 and masking the miRNA-485-5p binding site [71,97]. This event can trigger an increase of both *BACE1* mRNA and protein levels, leading to enhanced Aβ formation (Figure 4) [118,142,143,144]. Finally, *BACE1-AS* can decrease the level of miR-132-3p, which plays an important role in synaptic plasticity and activation [106,143].

*BC200* (brain cytoplasmic 200 RNA) (Table 1) is responsible for the synthesis of dendritic neural proteins and for long-term synaptic plasticity regulation by targeting eukaryotic initiation factor 4A (eIF4A). In AD, this transcript becomes upregulated with aging and disease progression with consequent alteration to the regulation of synaptic and dendritic transport through the microtubules, which leads to their degeneration [26,106,109,113,114,145].

Another interesting example of lncRNA linked to AD pathophysiology is the product of the sortilin receptor 1′s (*SORL1*) first intron, regulated byalternative splicing (*51A*) (Table 1). It has been reported that in AD patients, *51A* is upregulated, along with the lower level of *SORL1* expression. *51A* regulates the alternative splicing of *SORL1* mRNA, downregulating the production of the canonical variant of this receptor. These events can drive AD pathophysiology, as *SORL1* has a neuroprotective property by binding to apolipoprotein E (APOE), which interacts with Aβ, reducing APP oligomerization in the *BACE1* amyloid pathway [109,142,146,147,148].

LncRNA *E230001N04Rik* has been shown to regulate tau aggregates production in AD in the okadaic-acid induced in vitro AD model. This happens due to the upregulation of *E230001N04Rik* lncRNA’s neighboring genes, which are responsible for tau’s production (*Sepk1*), stability and aggregation (*Fkbp5*). Both of these genes are upregulated in AD patients. Moreover, the KD of this lncRNA in the HT22 cell line resulted in significantly lower tau production compared to the control [136].

LncRNAs *BDNF-AS* and *GDNF-OS* are, respectively, the antisense transcripts of brain-derived neurotrophic factor (*BDNF*) and glial cell line-derived neurotrophic factor (*GDNF*). They both negatively regulate neurotrophic factor expression, promoting the pathogenesis of AD. In fact, low levels of *BDNF* and *GDNF* cause a minor neuroprotective effect against Aβ accumulation [29,149,150].

### 3.2. Amyotrophic Lateral Sclerosis (ALS)

**Figure 5:**
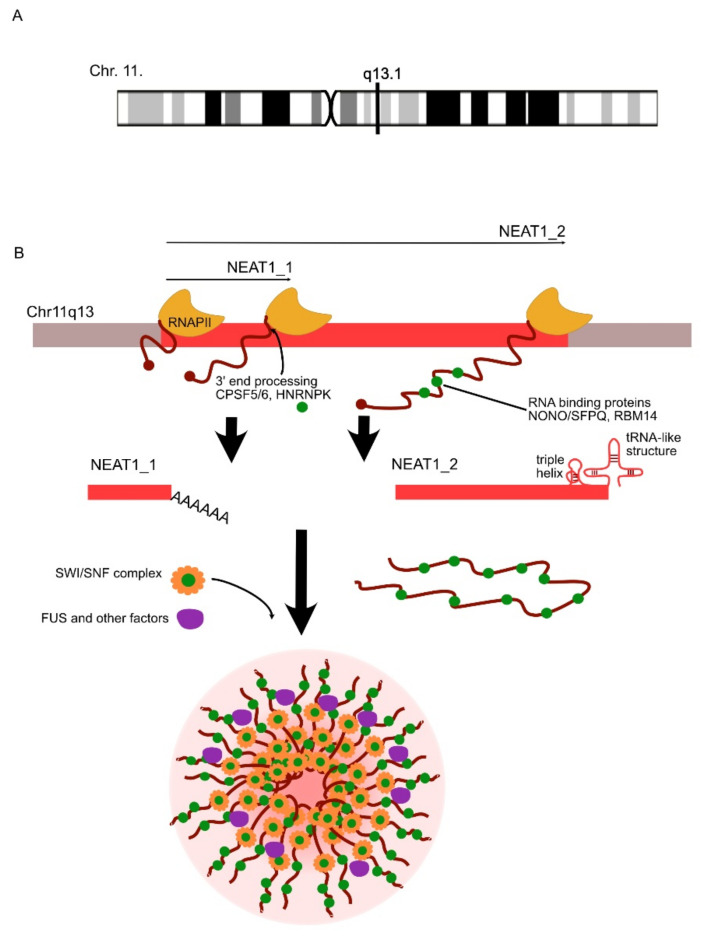
*NEAT1* in paraspeckle formation. (**A**) The *NEAT1* locus is localized on chromosome 11 (www.genecards.org, accessed on 28 April 2021). (**B**) *NEAT1* can be transcribed by the RNAPII into *NEAT_1* or *NEAT_2* transcripts, which vary in the structure of their 3′ end. The dot at the 5′ end of each variant represents the structure of a cap. The triple helix and tRNA-like structure at the 3′-end of *NEAT1_2* prevent the polyadenylation at the 3′-end of this isoform. HNRNPK (heterogeneous nuclear ribonucleoprotein K) is a crucial factor for *NEAT1_2* processing, as it prevents *NEAT1_1* polyadenylation, which is dependent on SPSF5/6. *NEAT1_2* additionally binds stabilizing PLD (phospholipase D)-containing, RNA-binding proteins such as NONO (non-POU domain-containing, octamer-binding protein), SFPQ (splicing factor, proline- and glutamine = rich) or RBM14 (RNA binding protein 14). The *NEAT1* lncRNA is localized into the cellular inclusions along with SWI/SNF (SWItch/sucrose non-fermentable) complexes (made out of grouped stabilizing, PLD-containing proteins) and FUS and other PLD-containing proteins.

ALS, also known as motor neuron disease (MND), is a severe neurodegenerative disorder that is not related to natural aging [151,152,153,154]. The progressive degeneration of neurons in ALS is observed in both upper and lower motor neurons. Depending on the affected region of the nervous system, ALS can result in various clinical symptoms, inevitably leading to muscle paralysis. The exact etiology is still unknown, and its pathophysiology can be quite diverse and involves mitochondrial dysfunction, the defective metabolism of RNA, disrupted axonal transport and misfolded protein aggregation [29,106,153,154]. As ALS has such diverse pathophysiology, there are still a lot of unknown mechanisms that may be driving its pathophysiology. LncRNAs have already been shown to play an important role in many of these mechanisms, especially in intracellular inclusion formation, which is a hallmark of ALS (Table 1) [106,155]. Some of these inclusions are stress granules (SGs) located in the endoplasmic reticulum; others are nuclear bodies (NBs) within the nucleus [49,156]. All of them are membranelle structures composed of RNAs and proteins, the formation of which takes place through liquid–liquid phase separation (LLPS) and is controlled by lncRNAs and RNA-binding proteins [156,157]. LLPS in healthy conditions is a reversible process wherein RNA low complexity domains, proteins and heterogeneous nuclear ribonucleoproteins interact and bind, forming droplet-like structures within the environment with liquid-like properties [126]. These structures are involved in homeostasis, but in ALS, due to increased cellular stress, they do not dissolve and lead to neurotoxicity. One of the key lncRNAs involved in their formation through LLPS is *NEAT1* (nuclear enriched abundant transcript 1) (Table 1), which regulates both SGs’ and NBs’ assembly and dynamics (Figure 5) [119,121,156,158,159]. *NEAT1* is most predominantly present in two forms: *NEAT1_1*, which regulates transcription by chromatin activation and *NEAT1_2*, which regulates the formation of paraspeckles (nuclear RNA granules formed through liquid–liquid phase separation). This second form is linked to ALS susceptibility [20,121]. In fact, in healthy mammals, generally *NEAT1_2* is not expressed, whereas in ALS patients, its level is increased. It can be found especially in the anterior horn of the spinal cord, leading to paraspeckle formation and further degeneration due to neurotoxicity [120,121]. Furthermore, *NEAT1* has been linked to the accumulation of misfolded TAR DNA-binding protein 43 (TDP-43) in intracellular inclusions. There is, in fact, a clear co-localization and binding of *NEAT1* and TDP-43 in cellular stress conditions, which can be found in cellular inclusions in some ALS cases [156]. Moreover, the same feature also underlies the pathophysiology of frontotemporal dementia (FTD), which is very often comorbid with ALS [106,151].

**Figure 6:**
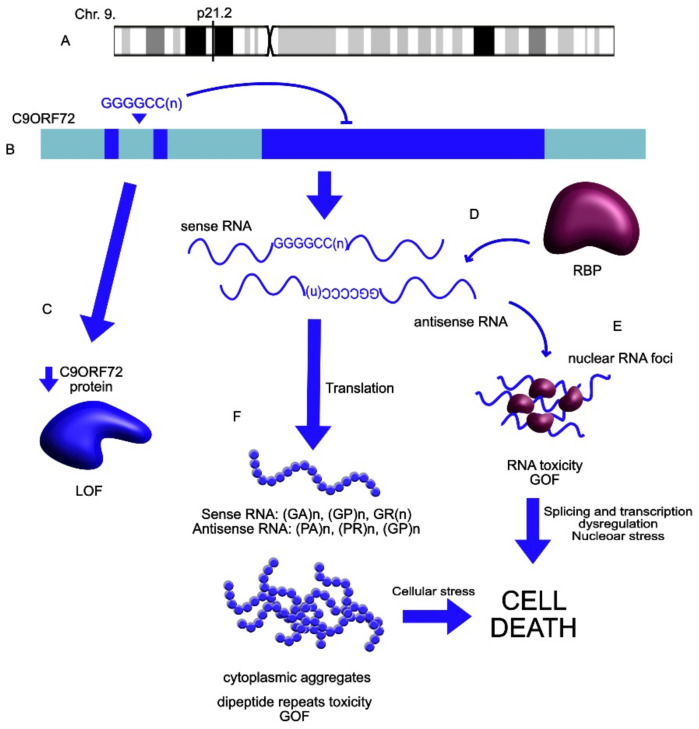
*C9ORF72*’s role in ALS pathophysiology. (**A**) The *C9ORF72* locus is localized on chromosome 9 (www.genecards.org, accessed on 28 April 2021). (**B**) In ALS patients, in *C9ORF72,* there have been observed a significantly extended number (>30) of GGGGCC repeats in the gene. (**C**) This mutation leads to impaired translation of the protein and its loss of function (LOF). (**D**) The sense and the antisense transcripts bind to RNA-binding proteins (RBP) forming nuclear foci and forming toxic aggregates (GOF: gain of function), which affects cellular processes such as splicing, transcription and nucleocytoplasmic transport and leads to nuclear stress (**E**,**F**). These transcripts are further translated into misfolded proteins, which can form toxic dipeptide aggregates (GOF). All the toxic cellular inclusions lead to cellular stress and eventually cell death (GA: glycine–alanine; GP: glycine–proline; GR: glycine-arginine; PA: proline–alanine; PR: proline–arginine; GP: glycine–proline).

*C9ORF72* is another example of an lncRNA associated with ALS (Table 1). It interacts with Rab proteins and controls endocytosis, autophagy and SG clearance. In ALS patients, it shows a significantly expanded number (>30) of GGGGCC repeats between 1a and 2b exons. After this transcript is translated, the protein loses its physiological function, which in healthy conditions, is linked to the regulation of endocytosis and autophagy [151,160,161]. Moreover, the extended number of hexanucleotide repeats of the sense and antisense RNA can co-localize with proteins involved in SGs formation, which is neurotoxic and leads to neurodegeneration (Figure 6) [124,158,162]. Additionally, the pathological repeats can be further translated into misfolded proteins, binding into toxic aggregates that can be found in the brain stem and spinal cord of some patients [158,163]. Finally, the repeat expansion can form a G-quadruplex structure that functions as a platform to recruit proteins such as TDP-43 and p62, forming pathological neuronal cytoplasmic inclusions [127]. *C9ORF72* can additionally undergo LLPS by forming droplet-like cellular inclusions [126,164].

*Sat III* (stress-induced satellite III repeat RNA) encodes for another lncRNA overexpressed in ALS (Table 1). Some studies of its functional orthologue in Drosophila melanogaster, *Hsrω*, showed that this transcript bound the protein dFUS, which was involved in ALS pathogenesis and could also be present in toxic cellular inclusions containing aggregated proteins [128,165]. The knockdown of this lncRNA led to dFUS translocation to the cytoplasm, altering the functioning of the protein [126,164]. Furthermore, *Hsrω* was also linked to TDP-43 aggregates formation, as it enhanced the expression of the gene encoding for this protein [166].

## 4. LncRNAs in Neurodevelopmental and Neuropsychiatric Disorders

LncRNAs play a key role in neurogenesis, synaptogenesis and brain development. Thanks to high-throughput technologies, it is clear that they are expressed in specific cell types, subcellular compartments and different regions of the brain [167,168]. Many lncRNAs are expressed in an age-dependent manner [169] and participate in neural cell fate determination [65]. Because of their involvement in these processes, any aberrant expression of these transcripts may result in neurodevelopmental or neuropsychiatric disorders such as autism spectrum disorder (ASD) or schizophrenia (SZ), among many others (Table 2) [65,170]. During neurodevelopment and brain functioning, GABAergic transmission is fundamental. Studies have shown that disrupted functions of GABAergic interneurons and associated lncRNAs can be observed in both ASD and SZ [170]. LncRNAs are also involved in many other genetic syndromes resulting in altered neurodevelopment including Angelman syndrome, Rett syndrome, fragile X chromosome and Down syndrome, which are linked with a predisposition to intellectual disability and ASD features [52,53,54,55,56,57,170,171]. Moreover, some of these syndromes can be comorbid with other neurodevelopmental disorders, suggesting that they may share pathophysiology and some molecular pathways [170,171].

### 4.1. Autism Spectrum Disorder

ASD is a heterogeneous neurodevelopmental disorder that arises from defects and aberrant gene expression during development. Its clinical symptoms include various repetitive stereotyped behaviors and also defects in communication (verbal and non-verbal) and reciprocal social interactions [65]. The etiology of ASD is extremely complex, including both genetic and environmental influences. Studies showed that there were mutations linked to ASD that could range from single-nucleotide variants to copy number variants and chromosomal abnormalities that affected both coding and non-coding genes [188]. Because there is a vast heterogeneity of genetic components in ASD, and the brain tissue of patients cannot be used for clinical diagnostic purposes, this pathology is still being studied, and the role of lncRNAs is still yet to be fully investigated (Table 2). Several groups have tried to identify the expression profiles of dysregulated mRNAs and lncRNAs in peripheral leukocytes from the blood of patients in order to analyze in which pathways these transcripts are involved [183,189]. The studies confirmed that many dysregulated lncRNAs were associated with regulatory homeobox-related genes (*HOXA* and *HOXB*), which might have confirmed their important role in ASD pathophysiology. Moreover, Wang and colleagues [183] suggested that lncRNAs involved in synaptic vesicle transport/signaling and long-term potentiation and depression play a major role in ASD. The analysis of post-mortem human brain tissue (prefrontal cortex (PFC) and cerebellum) identified 222 aberrantly expressed lncRNAs in individuals with ASD compared to the control group. Moreover, there was relative homogeneity of lncRNA expression between the PFC and cerebellum in the post-mortem tissue of individuals with ASD compared to the control [55]. Most of these transcripts were associated with the protein-coding genes that were expressed throughout neurogenesis and brain development [190,191,192]. An example is the gene coding of the ubiquitin protein ligase E3A (*UBE3A*), which is also disrupted in Angelman syndrome, which shares some clinical symptoms with ASD [109,170,193,194,195]. Thirty-eight lncRNAs have been identified as the antisense transcripts to protein-coding genes already linked with ASD such as *SYNGAP1-AS*, which is shown to be upregulated in the post-mortem PFC of individuals with ASD [3,65,196].

Other studies showed that a certain single nucleotide polymorphism in the antisense transcript to a processed pseudogene of moesin (*MSNP1AS*) also could be present and upregulated in some ASD cases [197]. This lncRNA regulates the expression of moesin, which is an important factor in neurons regulating the immune response and architecture of the nucleus [3,65,198,199]. Upregulation of *MSNP1AS* leads to downregulation of moesin translation, negatively affecting neurite morphology and function [65,170,198,199].

*SHANK2-AS*, the antisense transcript of SH3 and multiple-ankyrin repeat-domains protein 2, and *BDNF-AS*, the antisense to the brain-derived neurotrophic factor, represent other examples of the antisense lncRNAs associated with ASD (Table 2) [183,200,201,202]. Most of these identified antisense transcripts were associated with regulatory homeobox-related genes (*HOXA* and *HOXB*), some of whose mRNAs were also found to be aberrantly expressed.

### 4.2. Schizophrenia

SZ is a developmental neuropsychiatric disorder affecting up to 1% of the population. This disease is characterized by positive symptoms such as delusions, hallucinations and psychosis and negative symptoms such as depression, apathy and dysphoria. The exact etiology of SZ is not yet known. However, it seems that there are strong influences from genetics, epigenetics and the environment [65,188,203].

**Figure 7:**
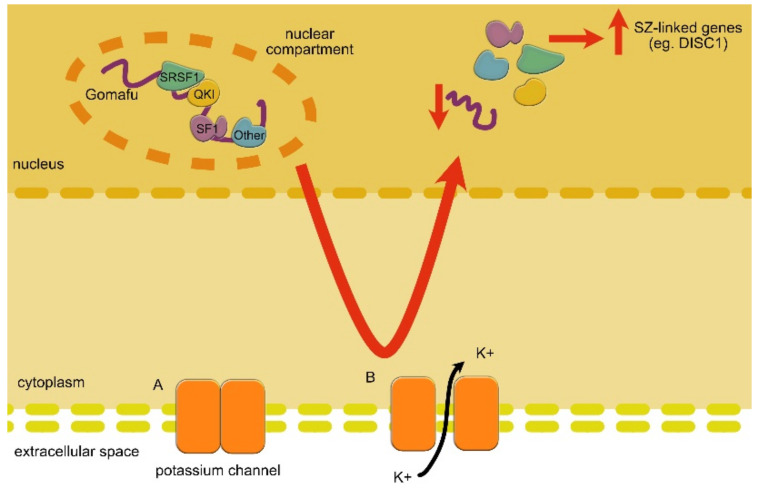
*GOMAFU* (*MIAT*) involvement in alternative splicing and SZ. When the cell is in an inactive state, the potassium channels (K+) are closed, and *GOMAFU* can bind several splicing factors (QKI; quaking homolog; SF1: splicing factor 1; SRSF1: serine/arginine rich SF1) involved in the expression of the SZ-related genes. In this way, it functional alternative splicing resulting in the decreased production of SZ-related proteins. When the cell becomes active, however, the ion channels open, and the influx of K+ ions leads to *GOMAFU* transcription downregulation (which is significantly decreased in SZ). The splicing factors are, therefore, free to act toward the alternative splicing of SZ-linked genes.

One of the lncRNAs most frequently linked with SZ is the nuclear transcript *GOMAFU* (Table 2) [23]. This lncRNA competitively binds to various miRNA and splicing factors, resulting in the decreased translation of SZ-linked mRNA [49,204]. In SZ post-mortem brain tissue, it was found that the *GOMAFU* expression level was significantly lower compared to the control. This downregulation has a very serious effect because it regulates splicing factors QKI (quaking homolog) and SRSF1 (serine/arginine-rich splicing factor 1), with global consequences on alternative splicing (Figure 7) [3,23,50]. It also upregulates two SZ-linked genes: *DISC1* (disrupted in schizophrenia 1) and *ERBB4* (v-erb-a erythroblastic leukemia viral oncogene homolog 4) [178,192,205]. *GOMAFU* knockdown results in splicing variants of these genes, similar to the ones observed in patients. Interestingly, the overexpression of *GOMAFU* in iPSCs resulted in the downregulation of the SZ-related splice variants of *DISC1* and *ERBB4*. This lncRNA has also been studied in the context of anxiety-related behavior (also occurring in SZ), as its levels are downregulated in the medial prefrontal cortex, impacting fear conditioning in mice. Mice with *GOMAFU* knockdown additionally present higher anxiety levels and fear-related behavior. While mice did not present any significant developmental abnormalities, they did present aberrations in their behavior and “wellbeing”, which might have suggested that even the slightest impact of *GOMAFU* function on neurodevelopment could result in significant behavioral changes [23,50,65,188,192]. Furthermore, *GOMAFU* takes part in the regulation of many other processes such as cell proliferation, migration and apoptosis through competitive interactions with various miRNAs [96,99,192,203].

The *DISC1-AS* lncRNA has been linked to SZ (Table 2), as it negatively regulates *DISC1* expression [65,188,192]. *DLG2AS* (antisense to discs large homolog 2) dysregulation has also been found in SZ patients’ brains. Through the control of *DLG2* gene expression (downregulated in the hippocampus of SZ patients), these lncRNAs impact brain development, long-term synaptic potentiation and axonal guidance signaling [188].

LncRNAs associated with the nuclear factor-κB (NF-κB) protein family have also been linked with SZ pathophysiology. This protein family has an important role in neurodevelopment, interacting with crucial genes/pathways for neurogenesis such as Notch, Shh and Wnt, and it also regulates inflammatory response [206]. In SZ patients, both NF-κB and genes that direct its translocation are downregulated, especially in the superior temporal gyrus. Safa and colleagues [206] showed that the dysregulated levels of lncRNAs associated with NF-κB could be found in the blood plasma of SZ patients. Most of these transcripts were upregulated in samples from the SZ group and might have been related to higher immune activation in the cortical regions. Moreover, most of these lncRNAs along the NF-κB pathway were involved in neurogenesis, synaptogenesis and brain development; hence, they may have played a role in the development of various neurological disorders including SZ [188,192,206].

## 5. Final Remarks

LncRNAs regulate several processes during brain development, neurogenesis, cell fate decision, maturation and differentiation [26] and are involved in cognitive mechanisms and memory formation [26]. Because lncRNAs act at different levels, any variation of their expression can result in developmental defects and neurological and psychiatric disorders [52]. One of the challenges in this field is to better understand the mechanisms of action of lncRNAs, the pathways in which they are involved and the partners with which they act during development. A more in-depth study of lncRNA and coding genes/genome architecture relationships could broaden the knowledge on the emerging link between neurodevelopmental defects and neurodegenerative phenotypes. Furthermore, understanding the roles of lncRNA-driven epigenetic modifications is also of pivotal importance for tailored treatments. It is therefore extremely necessary to develop adequate genetic tools and establish animal and in vitro models to study in detail the networks of lncRNAs and their alterations in disease. To date, only a small percentage of lncRNAs have been studied in pathological processes. With our review, we aimed to further stimulate lncRNA research in neurological and neurodevelopmental disorders for a broader understanding of disease and for implementing novel, RNA-based therapeutic approaches.

## Figures and Tables

**Table 1:** LncRNAs dysregulated in neurodegenerative disorders. The chosen examples of lncRNAs involved in neurodegenerative disorders. (UP: upregulation; DOWN: downregulation; references are also shown).

LncRNA	Expression Change	Role	Linked Disorder	References
*BACE1-AS*	UP	*BACE1-AS* enhances mRNA *BACE1* stability and activity. Leads to Aβ formation through the amyloid pathway.	AD	Faghihi et al., 2008 [97]; Faghihi et al., 2010 [71]
*51A*	UP	*51A* upregulates SORL1 variant A, which leads to Aβ accumulation through altered amyloid processing.	AD	Ma et al., 2009 [107]; Ciarlo et al., 2013 [108]; Luo and Chen 2016 [109]
*17A*	UP	*17A* disrupts GABAergic signalling (through the inhibition of GABAB R2 activity). This leads to an inflammation response and upregulation of Aβ formation and increases the Aβx-42/Aβx-40 ratio.	AD	Massone et al., 2011 [110]; Gavazzo et al., 2013 [111]; Buggia-Prevot and Thinakaran, 2014 [112]
*NDM29*	UP	*NDM29* promotes the cleavage activity of γ-secretase and *BACE1* secretase, increasing the production of Aβ formation and the Aβx-42/Aβx-40 ratio. Moreover, it triggers an inflammatory response.	AD	Massone et al., 2012 [113]
*BC200*	UP	*BC200* takes part in the maintenance of long-term synaptic plasticity by targeting eIF4A and interacting with local proteins. In AD, it leads to the increased loss of synapses.	AD	Mus et al., 2007 [114]; Lin et al., 2008 [115]
*NAT-Rad18*	UP	*NAT-Rad18* increases the cell death rate in neurons, promoting the apoptotic processes.	AD	Iacoangeli et al., 2010 [116]; Massone et al., 2012 [113]; Luo and Chen, 2016 [109]
*NEAT1*	UP/DOWN	*NEAT1* is involved in the decreased clearance of Aβ.	AD	Wang et al., 2019 [117]; Zhao et al., 2019 [118];
	UP	*NEAT1* regulates the assembly of paraspeckles and might trigger neurotoxic processes in ALS.	ALS	Clemson et al., 2009 [119]; Suzuki et al., 2019 [120]
*MALAT1*	UP	*MALAT1* facilitates paraspeckle formation by binding with FUS and TDP-43. Moreover, it controls the phosphorylation of SR proteins and gene expression *in cis*.	ALS	Clark et al., 2014 [50]; An et al., 2019 [121]; Wu and Kuo, 2020 [106]
*C9ORF72*	UP	*C9ORF72* extended repeats mutation leads to the repeat-associated translation into neurotoxic misfolded proteins and dipeptides. Contributes to the SGs’ formation and cellular inclusions.	ALS	Mizielinska et al., 2014 [122]; Wen et al., 2014 [123]; Maharjan et al., 2017 [124]; Wan et al., 2017 [29]; Swinnen et al., 2018 [125] Bampton et al., 2020 [126] Mizielinska et al., 2013 [127]
*SATIII*(*Hsrω*)	UP	*SAT III* binds to TDP-43 and takes part in promoting its elongation (by binding to the ELL2 domain) during the transcription, which can affect TDP-43 neurotoxicity.	ALS	Chung et al., 2018 [128]; Chen, K. and Chen, 2020 [5]; Wu, et al., 2020 [106]
*ATXN2-AS*	DOWN	*ATXN1-AS* extended repeats form RNA foci and lead to an increase in apoptosis through interactions with caspase 3/7.	ALS	Li et al., 2016 [129]
*SNAP25-AS*	DOWN	*SNAP25AS* affects *SNAP25* and processes controlled by it such as synaptic vesicle transport or axonal repair processes.	ALS	Gagliardi et al., 2018 [130]; Wu et al., 2020 [106]

**Table 2:** LncRNAs dysregulated in neurodevelopmental and neuropsychiatric disorders. The chosen examples of lncRNAs are involved in neurodevelopmental and neuropsychiatric disorders. (UP: upregulation; DOWN: downregulation; references are shown).

LncRNA	Expression Change	Role	Linked Disorder	References
*GOMAFU*	DOWN	*GOMAFU* controls alternative splicing through interactions with splicing factors. Moreover, it affects the specification of amacrine cells and is involved in SZ-related eye movement disorder.	SZ	Takahashi et al., 2003 [172]; Rapicavoli et al., 2010 [173]; Tsuiji et al., 2011 [95]; Ip et al., 2016 [174]
*MALAT1*	DOWN	*MALAT1* controls the expression of genes linked to synaptogenesis through interactions with SR proteins. The downregulation of *MALAT1* leads to impaired formation of synapses and reduced synaptic density.	SZ	Bernard et al., 2010 [175]; Madabhushi et al., 2015 [176]
*DISC1-AS*	DOWN	*DISC1-AS* affects cAMP signaling through interactions with DISC1 and DISC2.	SZ	Millar et al., 2004 [177]; Chubb et al., 2008 [178]
*DISC2-AS*	DOWN	*DISC2-AS* affects cAMP signaling (through interactions with DISC2), neuregulin signalling, axonal signalling and also long-term synaptic potentiation.	SZ	Polesskaya et al., 2003 [179]; Walsh et al., 2008 [180]
NEAT1	DOWN	*NEAT1* takes part in the unfolded proteins’ response under a condition of cellular stress.	SZ	Nakagawa et al., 2011 [181]; Hirose et al., 2014 [182]
*SHANK2-AS*	DOWN	*SHANK2-AS* affects the processes regulating post-synaptic density through interactions with SHANK2.	ASD	Wang et al., 2015 [183]
*BDNF-AS*	DOWN	*BDNF-AS* inhibits the BDNF transcript, which is a crucial transcription factor involved in neurite functioning.	ASD	Wang et al., 2015 [183]
*PTCHD1AS1-3*	DOWN	*PTCHD1AS1-3* is linked to the dysfunction of synapses and neurons in ASD.	ASD	Noor et al., 2010 [184]
*NRON*	UP	*NRON* inhibits NFAT (nuclear factor-activated T-cell) signaling.	Major Depressive Disorder	Willingham et al., 2005 [85]
*AK081227*	UP	*AK081227* downregulates GABAergic signalling through the inhibition of Gabrr2 expression.	Rett Syndrome	Petazzi et al., 2013 [54]
*Ube3aATS*	DOWN	*Ube3aATS* downregulation is associated with impaired contextual fear behavior (in Angelman syndrome). This is due to the impaired silencing of paternal Ube3a.	Angelman Syndrome	Meng et al., 2012 [185]; Meng et al., 2013 [186]; Meng et al., 2015 [187]
